# Development and Validation of Pregabalin in Bulk, Pharmaceutical Formulations and in Human Urine Samples by UV Spectrophotometry

**Published:** 2009-06

**Authors:** Rajinder Singh Gujral, Sk Manirul Haque, Prem Shanker

**Affiliations:** *Vardhman Chemtech Limited, Nimbua, Dera Bassi, Mohali, Punjab, India*

**Keywords:** pregabalin, validation, bulk drug, pharmaceutical formulations, human urine samples, uv

## Abstract

A simple and sensitive UV spectrophotometric method was developed and validated for the determination of pregabalin in bulk, pharmaceutical formulations and in human urine samples. The method was linear in the range of 0.5–5.0 μg/ml. There is no generally accepted method for the determination of pregabalin. The absorbance was measured at 210 nm. The method was validated with respect to accuracy, precision, specificity, ruggedness, and robustness, limit of detection and limit of quantitation. This method was used successfully for the quality assessment of five pregabalin drug products and in human urine samples with good precision and accuracy. This is found to be simple, specific, precise, accurate, reproducible and low cost UV Spectrophotometric method.

## INTRODUCTION

**Figure 1:**
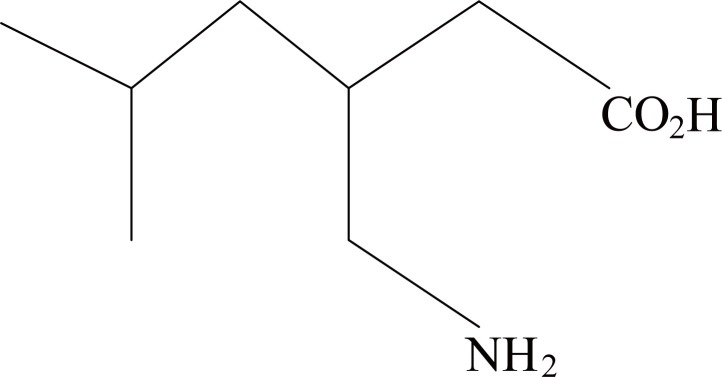
Structure of Pregabalin.

Pregabalin (PGB) is a new active substance known chemically as (S)–3–amino methyl–5–methyl hexanoic acid and is structurally related to the naturally occurring amino acids L – leucine and gamaa aminobutyric acid (GABA) (Fig. 1). It is a white to off – white crystalline, non – hygroscopic and water soluble (freely soluble below pH–3.7) powder. It contains one chiral centre, but is synthesized as the single enantiomer S. PGB exists as a single anhydrous and not solvated crystal form.

**Figure 2:**
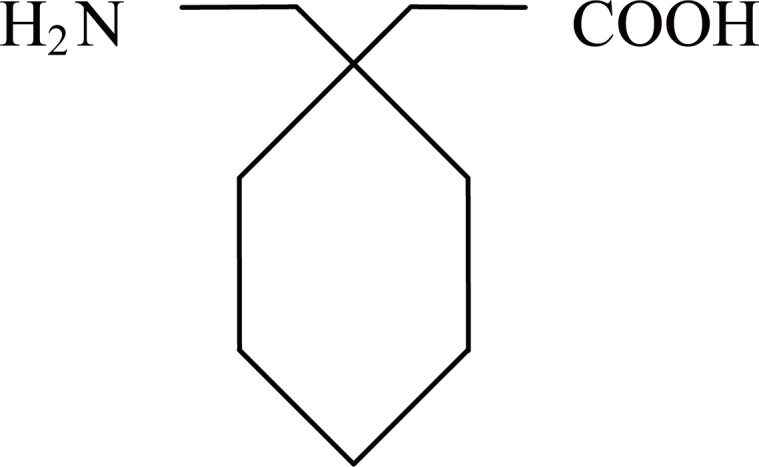
Structure of Gabapentin.

PGB is a new anticonvulsant and analgesic medication that was recently approved for adjunctive treatment of partial seizures in adults (1–4) in both the United States and Europe and for the treatment of neuropathic pain from postherpetic neuralgia and diabetic neuropathy. It is both structurally and pharmacologically related to the anticonvulsant and analgesic medication gabapentin (Fig. 2).

The mechanism of action is still unclear, pregabalin decreases central neuronal excitability by binding to an auxiliary subunit (α_2_–δ protein) of a voltage – gated calcium channel on neurons in the central nervous system. PGB reduces the release of several neurotransmitters include glutamate, norepinephrine, Substance P, and calcitonin gene related peptide from certain brain tissues and also reduce calcium influx in synaptosomes.

Pregabalin undergoes minimal metabolism in human with unchanged parent representing the majority (≥90 %) of drug – derived material (5). This contrasts with gabapentin, which is absorbed via a capacity limited L – amino acid transport system from the proximal small bowel into the blood stream (6–7).

The therapeutic importance of Pregabalin was behind the development of numerous methods for its determination. The methods adapted to the analysis of PGB include high – performance liquid chromatography (HPLC) (8), liquid chromatography – mass spectrophotometry (LC–MS) (9–10) and spectrofluorimetry (11). In addition, these methods require long and tedious pretreatment of the samples and laborious clean up procedures prior to analysis. An official monograph of PGB does not exist in any pharmacopoeia and determination of PGB in bulk and pharmaceutical formulations has not been yet described. Therefore, it is very imperative to develop a simple and suitable analytical method for the determination of PGB in bulk and pharmaceutical formulations. UV – Visible spectrophotometry is the technique of choice in research laboratories, hospitals and pharmaceutical industries due to its low cost and inherent simplicity.

This paper reports a simple, sensitive and accurate spectrophotometric method for the determination of PGB. The method is based on the direct measurement of native absorbance of the drug at 210 nm against the reagent blank. The proposed method was extended to the determination of PGB in bulk, pharmaceutical formulations and in human urine samples.

## EXPERIMENTAL

### Apparatus

Spectral runs were made on UV 3000^+^ UV/VIS spectrophotometer (LABINDIA^®^, Mumbai, India) with 1 cm matched glass cell.

### Materials and Reagents

- Pregabalin (Vardhman Chemtech Ltd, Punjab, India) was used as working standard.
- Pharmaceutical formulations of PGB such as Gabanext 75 (Nicholas Piramal India Ltd, Mumbai, India), Pregalin 75 (Torrent Pharmaceutical Ltd, Baddi, India), Neugaba 75 (Sun Pharmaceutical Industries, Jammu, India), Mahagaba 75 (Mankind Pharma Ltd, New Delhi, India) and Maxgalin 75 (Sun Pharmaceutical Industries, Jammu, India) were purchased from local markets.
- Sodium carbonate was purchased from Qualigens fine chemicals (Mumbai, India).
- Sodium bicarbonate was purchased from Qualigens fine chemicals (Mumbai, India).
- Urine samples were obtained from healthy volunteers.
- Carbonate buffer of pH 9.4 was prepared by dissolving 26.5 gm sodium carbonate and 21.0 gm sodium bicarbonate in 500 ml distilled water.

### Determination of appropriate UV wavelength

A suitable wavelength was required for the determination of Pregabalin. The appropriate wavelength for the determination of PGB was determined by wavelength scanning over the range 190–450 nm with a UV 3000^+^ UV/VIS spectrophotometer (LABINDIA^®^, Mumbai, India).

### Standard PGB Solution

A stock solution of PGB (50 μg/ml) was prepared by dissolving 5 mg PGB in 100 ml volumetric flasks with double distilled water. The stock solution (50 μg/ml) was used to prepare the working solutions by suitable dilutions with distilled water. The solutions were stable at least 10 days in room temperature.

## METHODS

### Procedure for the determination of PGB

**Figure 3:**
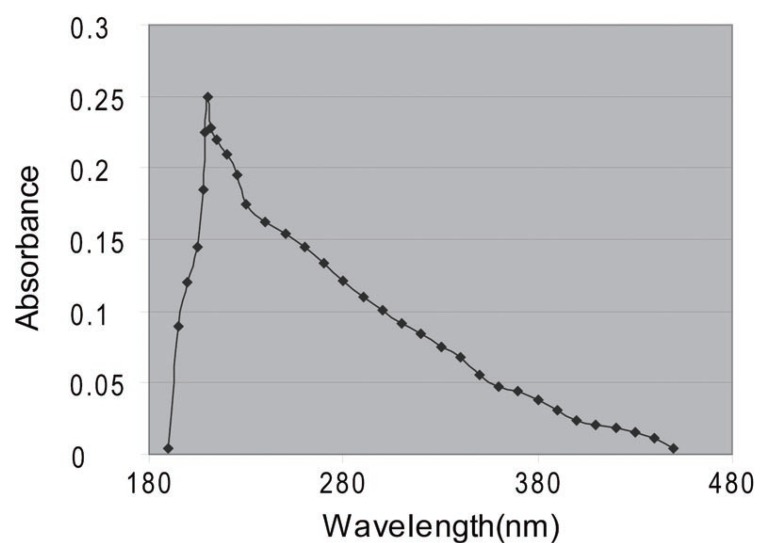
Absorption spectra of Pregabalin (5.0 μg/ml).

Aliquots of stock solution (50 μg/ml) were transferred into a set of 50 ml volumetric flasks and volumes were completed to the mark with distilled water to produce solutions in the concentration range 0.5–5.0 μg/ml. Absorbance was measured at 210 nm (Fig. 3) against the reagent blank. Calibration graphs were constructed by plotting absorbance against the final concentration of PGB.

### Procedure for determination of PGB in pharmaceutical formulations

One capsule (claiming 75 mg of Pregabalin) was accurately weighed and finely powdered. A quantity of the powder equivalent to 5 mg of PGB was extracted by shaking with 20 ml of distilled water, followed by another two extractions each with 10 ml distilled water. After passing through a 0.45 μm Millipore filter, the solution was diluted with distilled water to obtain a concentration of about 50 μg/ml. It was further diluted according to the need and then analyzed following the proposed procedures. The nominal content of the capsule was calculated either from the previously plotted calibration graphs or using regression equation.

### Procedure for determination of PGB in human urine samples

Aliquot volumes of human urine samples were transferred into small separating funnel. 10 ml of carbonate buffer pH–9.4 was added and solution was mixed well. The solution was then extracted with 2 × 10 ml diethyl ether. The ether extract was collected and evaporated. The residue was dissolved in 10 ml distilled water and above general procedure was then followed. The amount of PGB was obtained from the calibration graphs or corresponding regression equation.

## METHOD VALIDATION

The method was validated for selectivity, linearity, precision, accuracy, recovery and stability according to the principles of the Food and Drug Administration (FDA) industry guidance (12). Validation of analytical procedures is a vital aspect not just for regulatory purposes, but also for their efficient and reliable long – term application. The ICH guidelines achieved a great deal in harmonizing the definitions of required validation parameters, their calculation and interpretation. It is the responsibility of the analyst to identify parameters which are relevant to the performance of given analytical procedure as well as to design proper validation protocols including acceptance criteria and to perform an appropriate evaluation. The International Conference on the Harmonization of the Technical Requirements for Registration of Pharmaceuticals for Human Use has harmonized the requirements in two guidelines (13, 14). The first one summarizes and defines the validation characteristics needed for various types of test procedures, the second one extends the previous test to include the experimental data required and some statistical interpretation. These guidelines serve as a basis worldwide both for regulatory authorities and industry and bring the importance of a proper validation to the attention of all those involved in the process of submission. Nowadays, the validation characteristics needed for the various test procedures and their general requirements are well understood. The essential question to be answered is on the suitability of the calibration mode to be used in the test procedure. It should be noted that in most cases only a qualitative statement is needed.

The stability of the working PGB sample solutions at room temperature was evaluated with the help of UV spectra. The specificity and selectivity of the proposed method was evaluated by estimating the amount of PGB in the presence of common excipients lactose monohydrate, corn, starch, talc and methyl cobalamin.

The linearity of the proposed method was constructed for Pregabalin reference standard solution by plotting concentration of the compound versus the absorbance. The linearity was evaluated by linear regression analysis, which was calculated by the least square regression method. The parameters LOD and LOQ were determined on the basis of response and slope of the regression equation. The accuracy and precision of the method was evaluated within the linear range based on the analysis of PGB reference standard samples and pharmaceutical formulations at 2.0, 3.5 and 5.0 μg/ml. Five independent analysis were performed at each concentrations level within one day (intraday precision) as well as for five consecutive days (interday precision). The accuracy was ascertained by recovery studies using the standard addition method. The proposed method was used for estimation of PGB from capsules after spiking with 50, 200 and 350 % additional pure drug. The amount of PGB was determined from the regression equation.

## RESULTS AND DISCUSSIONS

In order to investigate the appropriate wavelength for the determination of PGB, solution of PGB was scanned by UV spectrophotometer in the range of 190 – 450 nm. The maximum absorbance was observed at 210 nm and this wavelength was fixed for the analysis of Pregabalin.

The absorbance – concentration plot for the proposed method was found to be rectilinear over the range of 0.5–5.0 μg/ml. Linear regression analysis of calibration data gave the regression equation cited in Table 1 with correlation coefficients close to unity. Statistical analysis of regression lines were made regarding the standard deviation of residuals (S_x/y_), standard deviation of slopes (S_b_) and standard deviation of intercepts (S_a_) and the values are summarized in Table 1.

**Table 1:** Summary of optical and regression characteristics of the proposed method

Parameters	Pregabalin

Linear dynamic range (μg/ml)	0.50–5.00
Regression equation^a^	Y = 5.02 × 10^−2^*X* – 1.8 × 10^−3^
S_a_	9.17 × 10^−4^
t S_a_^b^	2.04 × 10^−3^
S_b_	2.62 × 10^−4^
t S_b_^b^	5.84 × 10^−4^
Correlation coefficient (r)	0.9999
LOD (μg/ml)	2.47 × 10^−1^
LOQ (μg/ml)	8.15 × 10^−2^
Variance (S_o_^2^) of calibration line	1.54 × 10^−6^

a With respect to Y = a + b X, where X is the concentration in μg/ml, Y is Absorbance;

b Confidence interval of the intercept and slope at 95% confidence level and ten degrees of freedom (t=2.228).

The within day precision assays were carried out through replicate analysis (n=5) of PGB corresponding to 2.0, 3.5 and 5.0 μg/ml. The interday precision was evaluated through replicate analysis of the pure drug samples for five consecutive days at the same concentration levels as used in within day precision. The results of these assays are reported in Table 2. As can be seen from Table 2 that the recovery and RSD values for within day precision were always lower than 100.057 % and 0.891 %; recovery and RSD values for interday precision were lower than 100.056% and 0.968 %. The precision results are satisfactory. The intraday and interday precision assays were also carried for PGB in pharmaceutical formulations. The results are summarized in Table 3. As can be seen from Table 3 that the recovery and RSD values were in the ranges 99.920 to 100.171 %; 0.258 to 0.968 % and 99.801 to 100.199 % ; 0.203 to 1.136 % respectively for intraday and interday precision.

**Table 2:** Summary of accuracy and precision results of the proposed method in pure form

Proposed methods	Amount (μg/ml)		RSD (%)	REC. (%)	SAE^b^	C.L.^c^
	Taken	Found ± SD^a^				

Intra day assay	2.00	2.000 ± 0.018	0.891	99.990	8.0 × 10^-3^	6.7 × 10^-2^
	3.50	3.502 ± 0.014	0.402	100.057	2.8 × 10^-3^	7.8 × 10^-3^
	5.00	4.999 ± 0.017	0.333	99.999	7.5 × 10^-3^	2.1 × 10^-3^
Inter day assay	2.00	1.999 ± 0.019	0.968	99.924	8.7 × 10^-3^	2.4 × 10^-2^
	3.50	3.502 ± 0.024	0.697	100.056	1.1 × 10^-2^	3.0 × 10^-2^
	5.00	4.996 ± 0.020	0.399	99.920	8.9 × 10^-3^	2.5 × 10^-2^

a Mean for 5 independent analyses;

b SAE, standard analytical error;

c C.L., confidence limit at 95% confidence level and 4 degrees of freedom (t=2.776).

**Table 3:** Summary of accuracy and precision results of the proposed method in pharmaceutical formulations

Proposed methods	Amount (μg/ml)		RSD (%)	REC. (%)	SAE^b^	C.L.^c^
	Taken	Found ± SD^a^				

Intra day assay						
Gabanext-75	2.00	2.000±0.018	0.891	100.000	0.0070	0.0221
Gabanext-75	3.50	3.498±0.017	0.477	99.943	0.0075	0.0201
Gabanext-75	5.00	4.999±0.017	0.333	99.999	0.0074	0.0206
Pregalin-75	2.00	1.999±0.006	0.258	99.980	0.0023	0.0064
Pregalin-75	3.50	3.498±0.017	0.476	99.943	0.0074	0.0207
Pregalin-75	5.00	4.996±0.032	0.630	99.920	0.0141	0.0391
Neugaba-75	2.00	1.999±0.011	0.569	99.970	0.0051	0.0142
Neugaba-75	3.50	3.502±0.031	0.899	100.057	0.0141	0.0391
Neugaba-75	5.00	5.004±0.023	0.454	100.080	0.0102	0.0282
Maxgalin-75	2.00	1.999±0.011	0.569	99.970	0.0051	0.0142
Maxgalin-75	3.50	3.506±0.026	0.741	100.171	0.0117	0.0322
Maxgalin-75	5.00	4.996±0.020	0.399	99.940	0.0089	0.0247
Mahagaba-75	2.00	1.999±0.019	0.968	99.924	0.0087	0.0240
Mahagaba-75	3.50	3.498±0.030	0.845	99.943	0.0132	0.0367
Mahagaba-75	5.00	5.000±0.022	0.436	100.000	0.0098	0.0271
Inter day assay						
Gabanext-75	2.00	1.996±0.011	0.547	99.801	0.0049	0.0136
Gabanext-75	3.50	3.494±0.018	0.510	99.829	0.0079	0.0221
Gabanext-75	5.00	4.996±0.024	0.488	99.920	0.0109	0.0303
Pregalin-75	2.00	1.999±0.011	0.569	99.970	0.0051	0.0142
Pregalin-75	3.50	3.499±0.012	0.341	99.967	0.0053	0.0172
Pregalin-75	5.00	4.997±0.010	0.203	99.940	0.0045	0.0125
Neugaba-75	2.00	2.004±0.016	0.832	100.199	0.0075	0.0020
Neugaba-75	3.50	3.498±0.012	0.331	99.943	0.0052	0.0144
Neugaba-75	5.00	4.997±0.013	0.264	99.940	0.0059	0.0164
Maxgalin-75	2.00	2.000±0.022	1.136	99.999	0.0102	0.0282
Maxgalin-75	3.50	3.498±0.017	0.476	99.943	0.0074	0.0207
Maxgalin-75	5.00	4.997±0.013	0.264	99.940	0.0059	0.0164
Mahagaba-75	2.00	2.000±0.018	0.891	100.000	0.0070	0.0221
Mahagaba-75	3.50	3.502±0.031	0.899	100.057	0.0141	0.0391
Mahagaba-75	5.00	4.997±0.010	0.203	99.940	0.0045	0.0125

a Mean for 5 independent analyses;

b SAE, standard analytical error;

c C.L., confidence limit at 95% confidence level and 4 degrees of freedom (t=2.776).

The proposed method was used for estimating of PGB from capsules after spiking with 50, 200 and 350 % of additional pure drug. The results are reported in Table 4. As can be seen from Table 4 that the recovery and RSD values were in the ranges 99.920 to 100.027 % and 0.152 to 0.923 %. The selectivity of the propose method was ascertained by analyzing standard PGB in the presence of excipients such as lactose monohydrate, corn, starch, talc and methyl cobalamin. It was observed that the excipients did not interfere with the proposed method.

**Table 4:** Summary of data for the determination of pregabalin in pharmaceutical preparations by standard addition method

Formulations	Amount (μg/ml)			Recovery (%)	RSD (%)	SAE^b^
	Taken	Added	Found ± SD^a^			

Capsules						
Gabanext-75	1.00	0.50	1.499 ± 0.008	99.920	0.503	0.0034
	1.00	2.00	3.001 ± 0.012	100.026	0.399	0.0054
	1.00	3.50	4.499 ± 0.011	99.982	0.233	0.0047
Neugaba-75	1.00	0.50	1.499 ± 0.004	99.973	0.238	0.0016
	1.00	2.00	2.999 ± 0.011	99.987	0.350	0.0047
	1.00	3.50	4.498 ± 0.008	99.965	0.176	0.0035
Maxgalin-75	1.00	0.50	1.500 ± 0.009	99.973	0.590	0.0040
	1.00	2.00	2.999 ± 0.014	99.960	0.474	0.0064
	1.00	3.50	4.500 ± 0.008	100.001	0.175	0.0035
Pregalin- 75	1.00	0.50	1.499 ± 0.014	99.947	0.923	0.0062
	1.00	2.00	2.999 ± 0.009	99.973	0.284	0.0038
	1.00	3.50	4.498 ± 0.010	99.946	0.220	0.0044
Mahagaba-75	1.00	0.50	1.499 ± 0.008	99.973	0.473	0.0032
	1.00	2.00	3.001 ± 0.007	100.027	0.242	0.0033
	1.00	3.50	4.496 ± 0.006	99.920	0.152	0.0031

a Mean for 5 independent analyses;

b SAE, standard analytical error.

The proposed method was further extended to the *in vitro* determination of PGB in spiked human urine samples. In neuropathic patients, PGB is orally given in doses of 150 to 600 mg per day, with an associated mean of around 123 μg.hr/ml. Pregabalin undergoes minimal metabolism in human with unchanged parent representing ≥90% of drug derived in urine. This concentration fell well within working range of proposed method. The calibration graphs were constructed by plotting absorbance versus increasing concentrations of PGB in spiked human urine samples over the concentration range 0.5–5.0 μg/ml. The results (Table 5) are satisfactorily accurate and precise.

**Table 5:** Application of the proposed UV method to the determination of pregabalin in human urine samples

Amount added (μg/ml)	Amount found (μg/ml)	Recovery (%)

1.00	0.9721	97.210
2.00	1.9681	98.405
3.00	2.9641	98.803
4.00	3.9801	99.503
5.00	4.9960	99.920
X		98.768
RSD		1.065

## CONCLUSIONS

The proposed method does not require any laborious clean up procedure before measurement. In addition, the method has wider linear dynamic range with good accuracy and precision. The method shows no interference from the common excipients and additives. Since in human unchanged parent representing ≥ 90% of drug is derived in urine, this method can be used for estimating unabsorbed PGB in urine samples by very simple, cost affective, fast and efficient method. This may help in analyzing affectivity of this drug in human beings during treatment. Therefore, it is concluded that the proposed method is simple, sensitive and rapid for the determination of pregabalin in bulk, pharmaceutical formulations and in human urine samples.
